# Safety Profile and Tumor Response of EGFR-TKIs in Clinical Practice: A Real-World Study in Thailand

**DOI:** 10.3390/jcm15062437

**Published:** 2026-03-23

**Authors:** Pattama Jainan, Chayanat Pongsathabordee, Kamala Sadabpod, Titima Junkrut, Thanakorn Jerasirichot, Oran Phetchuensakun, Taniya Paiboonvong, Saranporn Srithonrat

**Affiliations:** 1 Department of Pharmacy, Rajavithi Hospital, Bangkok 10400, Thailand; jainanpatm@gmail.com; 2 Department of Pharmacy Practice, College of Pharmacy, Rangsit University, Mueang Pathum Thani 12000, Thailand; chayanat.p@rsu.ac.th (C.P.); kamala.s@rsu.ac.th (K.S.); titima.j63@rsu.ac.th (T.J.); thanakorn.j63@rsu.ac.th (T.J.); oran.p63@rsu.ac.th (O.P.); 3 Faculty of Pharmacy, Pathumthani University, Mueang Pathum Thani 12000, Thailand

**Keywords:** epidermal growth factor receptor tyrosine kinase inhibitors, adverse events, erlotinib, gefitinib, osimertinib

## Abstract

**Background**: Epidermal growth factor receptor tyrosine kinase inhibitors (EGFR-TKIs) are the first-line treatment for patients with non-small cell lung cancer (NSCLC) harboring EGFR mutations. Although EGFR-TKIs can cause various adverse events (AEs), their profiles have not been fully elucidated in Thai patients. This study aimed to determine the incidence, characteristics, severity, and duration of the first AEs and to evaluate their association with tumor response in patients with NSCLC receiving EGFR-TKIs. **Method**: This retrospective cohort study was conducted at a super-tertiary care hospital in Thailand. Patients with NSCLC who received EGFR-TKIs between August 2021 and July 2024 were included. Descriptive statistics were used to summarize safety profiles and tumor response. The association between AEs and objective response was assessed using logistic regression. **Results**: A total of 187 patients were included in this study. Overall, 177 AEs were observed in patients receiving erlotinib, osimertinib, or gefitinib. The most common cutaneous AEs were rash (30.7%), xerosis (24.1%), and acneiform rash (19.3%), while diarrhea (20.3%) was the most frequent gastrointestinal toxicity. Most AEs were grade 1–2 and occurred within 1 month after treatment initiation. In multivariable logistic regression analysis, pruritus (OR 8.26, 95% CI: 1.00–67.75, *p* = 0.049) and treatment line (OR 0.27, 95% CI: 0.10–0.68, *p* = 0.006) were independently associated with objective response. **Conclusion**: Most of the AEs occurred early during EGFR-TKI therapy, with cutaneous reactions being the most common and generally mild to moderate. Pruritus and treatment line were independently associated with objective response, suggesting that pruritus may serve as a potential clinical indicator of treatment response and highlighting the importance of monitoring of the EGFR-TKI-related AEs during therapy.

## 1. Introduction

Epidermal growth factor receptor tyrosine kinase inhibitors (EGFR-TKIs) have been recommended by the American Society of Clinical Oncology (ASCO), the European Society for Medical Oncology (ESMO), and the National Comprehensive Cancer Network (NCCN) as a first-line treatment for patients with EGFR-positive NSCLC. The EGFR mu-tations are found in approximately 10% of white patients with NSCLC and up to 19% of Black and 50% of Asian patients [1,2,3,4]. Due to their mechanisms of action, EGFR-TKIs result in both tumor suppression and treatment-related toxicities. The treatment with EGFR-TKIs can cause adverse events (AEs) that may affect quality of life and lead to treatment discontinuation. EGFR is mainly expressed in epithelial cells, such as the skin and gastrointestinal tract. Therefore, cutaneous and gastrointestinal reactions are frequently reported during EGFR-TKI therapy [5,6,7]. A recent meta-analysis reported that the most common of all grade AEs were diarrhea (53.7%), rash (48.6%), and mucositis (46.5%) [8]. Adverse effects in patients treated with EGFR-TKIs vary across the study. However, the characteristics and clinical implications of these adverse events in real-world clinical practice, particularly in Asian populations, remain unclear.

Importantly, emerging evidence suggests that certain AEs may reflect the pharma-codynamic activity of EGFR-TKIs and could be associated with treatment efficacy. Several studies have reported that patients who develop EGFR-TKI-related toxicities, particularly cutaneous toxicities such as rash, may experience improved clinical outcomes compared with those without these events. The severity of rash has also been associated with better outcomes [9,10,11]. Gastrointestinal toxicity such as diarrhea have also been reported to correlate with treatment efficacy, with one study identifying diarrhea as an independent predictor of tumor response [12]. Nevertheless, few studies have examined EGFR-TKI-related AEs and their association with tumor response in Thailand. Therefore, this study aimed to determine the incidence, characteristics, severity, and duration of the first AEs and to evaluate their association with tumor response in patients with NSCLC receiving EGFR-TKIs.

## 2. Methods

### 2.1. Study Design and Participants

This retrospective cohort study was conducted at Rajavithi Hospital, a super-tertiary care hospital in Thailand. Adult outpatients (age > 18 years) who were diagnosed with NSCLC and treated with EGFR-TKIs between August 2021 and July 2024 were included in the follow-up until September 2024. Patients who were lost to follow-up or died without documented causes and therefore had no evaluable tumor response data were excluded from the outcome analysis.

### 2.2. Data Collection

The data were collected from electronic medical records, including demographic data, subtype of NSCLC, treatment line of EGFR-TKIs, characteristics of AEs, grading severity, duration from drug initiation to the first AEs, and tumor response.

First-line treatment was defined as patients receiving EGFR-TKIs after diagnosis with NSCLC, and second-line treatment was defined as patients receiving EGFR-TKIs after being treated with one of standard chemotherapy formula.

The severity of AEs was graded according to the Common Terminology Criteria for Adverse Events (CTCAE), version 5.0; grade 1 (Mild), grade 2 (Moderate), grade 3 (severe or medically significant but not immediately life-threatening), grade 4 (life-threatening consequences; urgent intervention indicated), and grade 5 (death related to AE) [13]. The CTCAE grading was not systematically implemented in routine clinical practice. The severity grades were recorded based on physician-documented assessments. If the severity was not specified, AEs were retrospectively graded based on the documented symptom characteristics and required medical interventions in accordance with the CTCAE criteria.

Tumor response was extracted based on physician-documented assessments in the medical records. Imaging evaluations were performed as part of routine clinical care, typically every 3 months, according to physician’s discretion and clinical status. According to Response Evaluation Criteria in Solid Tumors (RECIST), version 1.1 [14], tumor response is categorized into four groups: complete response (CR), defined as the disappearance of all target lesions; partial response (PR), defined as at least a 30% decrease in the sum of diameters (SOD) of target lesions; progressive disease (PD), defined as at least a 20% increase in SOD or the appearance of new lesions; and stable disease (SD), defined as neither sufficient shrinkage to qualify for PR nor sufficient increase to qualify for PD. Formal RECIST measurements were not systematically implemented in routine practice. Tumor responses were categorized as objective response (CR or PR) or non-response (SD or PD).

### 2.3. Statistical Analysis

Descriptive statistics were used to summarize baseline characteristics and the incidence of AEs. Categorical variables were presented as frequencies and percentages, while continuous variables were presented as mean ± standard deviation (SD). Categorical variables were compared using the chi-square test or Fisher’s exact test, as appropriate. For the analysis of AE incidence, percentages were calculated based on the total number of treatment episodes (*n* = 212) to capture drug-specific toxicities. Treatment episodes were analyzed separately for each drug. The patients who switched between EGFR-TKIs contributed to more than one treatment episode, and each episode was considered a separate observational unit for analysis. For tumor response analysis, CR and PR were considered as objective response, and PD and SD were considered as non-responses.

The association between AEs and objective response was evaluated using logistic regression analysis. Univariate logistic regression was performed to assess the association between each variable and objective response. Variables with very low frequency were excluded from the regression analyses due to the risk of unstable estimates. Furthermore, clinically relevant AEs, including rash, xerosis, acneiform rash, papulopustular rash, pruritus, paronychia, mucositis, and diarrhea, were evaluated in the univariable analysis. In addition, baseline variables, including age, treatment line, and EGFR-TKI generation were assessed as potential predictors of the response.

Variables with a *p*-value of < 0.20 in the univariable analysis were entered into the multivariable logistic regression model to control for potential confounders and to identify independent predictors of tumor response. Potential collinearity among variables was assessed using the variance inflation factor (VIF), with values >5 indicating significant col-linearity. Adjusted odds ratios (aORs) with 95% confidence intervals (CIs) were reported. Statistical significance in the final model was defined as a *p*-value of < 0.05.

Descriptive statistics were performed using IBM SPSS Statistics (version 25.0), while logistic regression analyses were conducted using Stata (version 16.1).

## 3. Results

A total of 187 patients were included in this study. Females were predominant (70.6%). The mean age was 66.37 ± 10.65 years. Almost all patients had stage IV NSCLC (95.7%), and most had adenocarcinoma (88.2%). Most patients had no smoking history (78.8%). The baseline characteristics of patients receiving EGFR-TKIs are summarized in Table 1. Among them, 24 patients received more than one EGFR-TKI during the study period. Erlotinib accounted for 76.9% of EGFR-TKI treatments and was predominantly used as a first-line therapy, whereas osimertinib was more frequently prescribed after dis-ease progression or intolerance to erlotinib.

### 3.1. Characteristics of Adverse Events

**Figure 1:**
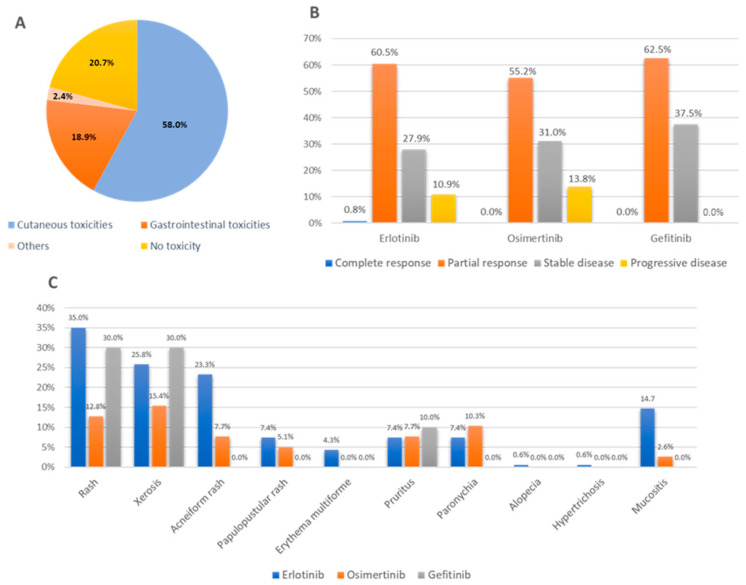
(**A**) Distribution of adverse events according to toxicity category. (**B**) Tumor response according to EGFR-TKI agents. (**C**) Frequency of specific adverse events stratified by EGFR-TKI agents.

We found that at least one AE occurred in 163 patients (87.2%). The majority of AEs were cutaneous in nature (87.0%). Diarrhea was the most common gastrointestinal toxicity (20.3%). The distribution of AEs by toxicity category is illustrated in Figure 1A. Among cutaneous toxicities, rash had the highest incidence (30.7%), followed by xerosis (24.1%) and acneiform rash (19.3%). The incidence and characteristics of AEs associated with EGFR-TKI therapy, including erlotinib, osimertinib, and gefitinib, is presented in Table 2. The proportion of cutaneous toxicities is shown in Figure 1C. The onset of the first event ranged from 1 week to 9 months, with most events occurring less within 1 month.

Most patients were able to tolerate reactions with grade 1–2 severity (Table 3). The severity of grade ≥ 3 resulted from rash, xerosis, acneiform rash, paronychia, mucositis, and diarrhea. There was no fatal toxicity found in this study. However, skin reactions and liver injury caused the drug discontinuation in three patients receiving erlotinib.

### 3.2. Tumor Response

Tumor response was assessed by oncologists in 166 of 212 treatment episodes (78.3%), while 46 episodes (21.7%) were excluded due to incomplete response documentation. Among the evaluable episodes, 137 (82.5%) had documented AEs, whereas 39 (84.8%) of the excluded episodes had recorded AEs. Tumor responses among evaluable treatments were as follows: CR was observed in 1 of 166 episodes (0.6%), PR in 99 (59.6%), SD in 48 (28.9%), and PD in 18 (10.8%). The objective response rate (CR or PR) was 60.2%. Tumor response according to EGFR-TKI agents is shown in Figure 1B.

In comparisons, pruritus was significantly associated with objective response (12% vs. 1.5%, *p* = 0.014). Rash also showed a higher proportion of response in patients who developed the event; however, the association did not reach statistical significance (*p* = 0.804). No significant associations were observed for other AEs (*p* > 0.05). The results are summarized in Table 4.

In univariable analysis, pruritus (OR 8.86, 95% CI: 1.12–69.89, *p* = 0.038) and treatment line (OR 0.27, 95% CI: 0.11–0.68, *p* = 0.005) were significantly associated with objective response. In multivariable analysis, both pruritus (OR 8.26, 95% CI: 1.00–67.75, *p* = 0.049) and treatment line (OR 0.27, 95% CI: 0.10–0.68, *p* = 0.006) remained significantly associated with objective response, while other variables were not significant (Table 5).

## 4. Discussion

This study provides the first comprehensive real-world data of AEs associated with EGFR-TKIs in Thai patients with NSCLC, extending beyond previous reports, including osimertinib-related AEs. Our findings indicate that EGFR-TKIs exhibited a high incidence of AEs. Overall, cutaneous reactions were the most common AEs observed with various clinical manifestations, with rash occurring most frequently. Most events occurred early after treatment initiation and were mild to moderate in severity. Similarly to the results of a network meta-analysis, approximately 50% of patients treated with EGFR-TKIs experienced AEs within 30 days, and rash had the highest incidence rate [15].

To the best of our knowledge, skin toxicity has been widely described in the literature and can be explained by the physiological role and widespread expression of EGFR in normal skin cells. EGFR-TKIs inhibit the EGFR signaling pathway not only in tumor cells but also in keratinocytes, sebaceous glands, and hair follicles. Furthermore, variability in the incidence and severity of skin reactions may be influenced by multiple factors, including gene polymorphisms and pharmacokinetic differences [16,17,18,19,20,21]. Thus, patients treated with different inhibitors may suffer from different dermatologic effects. We observed AEs from osimertinib was lower than erlotinib, and grade 3 or higher was not found. Newer-generation of EGFR-TKIs were noted as fewer dermatological reactions by spare wild-type EGFR. The different toxicity profiles between first/second/third-generation EGFR-TKIs were reported that the likelihood of causing grade ≥3 AEs of osimertinib lower than erlotinib and gefitinib [22]. Moreover, osimertinib generally demonstrates a milder toxicity profile, particularly for dermatologic, nail, and hair toxicities, with a lower rate of grade ≥3 compared with gefitinib or erlotinib, especially in Asian patients [23].

Gastrointestinal toxicities, especially diarrheas, are one of the most common events reported with 20–96% of patients, resulting in dose reduction and treatment discontinuation. The possible mechanisms for EGFR-TKIs-induced diarrhea have been proposed to include secretory mechanisms, histopathological changes (impaired epithelium generation and barrier leakage), inflammation, and alterations in the gut microbiota [24,25]. In line with our study, diarrhea was commonly found in 20.3% of patients and caused severe symptoms in one patient treated with loperamide.

The clinical features of AEs in our study are consistent with previous reports from Thailand, where the incidence of cutaneous toxicity was high. The study by Rongngern et al. found that most of cutaneous reactions of EGFR-TKIs were xerosis (65.9%), paronychia (57.4%), and papulopustular rash (42.6%). The onset was found within 1–3 months [26]. Chanprapaph et al. reported that approximately 70% of Thai patients developed cutaneous AEs; they occurred with erlotinib in 70.7% and gefitinib in 62.5% of patients, and most of the events included xerosis (52.5%) [27]. Chularojanamontri et al. studied cutaneous AEs of EGFR inhibitors in Thai patients. The three most common skin reactions were xerosis, acneiform rash/papulopustular eruption, and pruritus caused by erlotinib or gefitinib. Most reactions occurred within 1 month [28]. Nevertheless, there was a slight difference compared to previous Thai studies, where xerosis was mostly found. This may be a result of many factors, such as genetic polymorphisms, immune system, and environmental factors [29,30,31,32]. Given Thailand’s tropical and hot climate, the higher occurrence of xerosis may be explained. However, rash commonly coexisted with xerosis in our study.

According to the proposed guidelines for managing EGFR-TKI-associated AEs, top-ical or oral corticosteroids and antibiotics have been recommended to manage cutaneous toxicity, depending on the severity. Dose reductions and treatment interruptions were the common strategies for managing severe adverse effects [7,33,34,35]. In our study, the treatment options varied based on the clinical presentation and were consistent with the recommended management strategies in which topical and oral corticosteroids were commonly used for rash management, including antibiotics when secondary bacterial infection is suspected. Xerosis was managed with moisturizers or urea-based creams, generally used with oral antihistamines to relieve pruritus. Moreover, emollients and sunscreen were advised for most patients.

Considering the relationship between AEs and objective response, In the multivariable analysis, pruritus was significantly associated with objective response. This finding is consistent with previous reports suggesting that certain cutaneous toxicities may reflect effective EGFR pathway inhibition [10,11,12,17,36]. Likewise, various cutaneous reactions have been reported in relationship in Thai patients—papulopustular rash, maculopapular rash, mucositis, paronychia, and trichomegaly [26,27,28]. Nevertheless, rash, acneiform rash, and papulopustular rash were not significantly associated with objective response in our study. Previous studies have suggested that the association between rash and treatment efficacy may be more evident in patients who develop moderate to severe rash [9,10,11]. In this study, most rash events were grade 1–2, while grade ≥3 rash was relatively uncommon, which may have attenuated the potential association with objective response. Treatment line was also found to be significantly associated with objective response. Patients receiving EGFR-TKIs as first-line therapy may achieve better outcomes due to greater tumor dependence on EGFR signaling before the development of resistance mechanisms.

In our cohort, approximately 20% of patients received EGFR-TKIs as second-line therapy after prior chemotherapy, and previous treatment exposure or more aggressive disease may have contributed to reduced responsiveness. Thus, our results suggest that specific cutaneous reactions, particularly pruritus, may serve as potential surrogate indicators of objective response. However, given the wide confidence intervals and the retrospective design, these findings should be interpreted with caution and warrant prospective validation.

EGFR-TKI-induced toxicities are a significant clinical challenge and may lead to treatment discontinuation and poor patient adherence. Early detection and appropriate management of adverse reactions are essential to prevent treatment interruption and to improve patients’ quality of life. Patient counseling is an important management method. Education regarding potential AEs and symptom management may benefit patients receiving EGFR-TKIs. In addition, pharmacist-led monitoring may represent a promising area for future implementation and research.

This study has several limitations. First, as a retrospective study, it is subject to inherent limitations, including potential selection bias and incomplete data from electronic medical records. Documentation bias may also have occurred because AEs and clinical outcomes were extracted from physician-recorded clinical notes. Second, this study was conducted at a single academic center and included only patients with NSCLC; therefore, the findings may not be generalizable to other healthcare settings or cancer populations. Third, although multivariable analysis was performed to adjust for measured confounders, residual or unmeasured confounding cannot be excluded. In addition, missing tumor response data may have affected the accuracy of outcome classification, which may have introduced selection bias. The relatively limited sample size may also have reduced the ability to detect associations with less frequent AEs. Despite these limitations, the findings provide clinically relevant insights into the relationship between EGFR-TKI-related AEs and objective response. Future prospective, multicenter studies with standardized out-come assessment are warranted to validate these findings.

## 5. Conclusions

Most of the AEs occurred early during therapy, with cutaneous reactions being the most common and generally mild to moderate, allowing EGFR-TKI therapy to be continued with appropriate management. Pruritus and treatment line were independently associated with objective response, suggesting that pruritus may serve as a potential clinical indicator. These findings emphasize the importance of monitoring EGFR-TKI-related AEs and supporting patient education to optimize treatment management.

## Figures and Tables

**Table 1:** Baseline characteristics of patients included in this study (*n* = 187).

Characteristics	Number of Patients (%)
Sex	
Female	132 (70.6)
Male	55 (29.4)
Age (mean ± SD)	66.37 ± 10.65
Subtype of NSCLC	
Adenocarcinoma	165 (88.2)
Squamous cell carcinoma	14 (7.5)
Others	8 (4.3)
Stage of NSCLC	
I	2 (1.1%)
II	2 (1.1%)
III	4 (2.1%)
IV	179 (95.7%)
Treatment line	
First-line treatment	167 (89.3)
Second-line treatment	20 (10.7)

**Table 2:** Characteristics of adverse events categorized by treatment episodes (*n* = 212).

	Treatment Episodes, *n* (%)			
	Overall (*n* = 212)	Erlotinib (*n* = 163)	Osimertinib (*n* = 39)	Gefitinib (*n* = 10)
Adverse events	177 (83.5)	145 (89.0)	26 (66.7)	6 (60)
Cutaneous toxicities				
Rash	65 (30.7)	57 (35.0)	5 (12.8)	3 (30.0)
Xerosis	51 (24.1)	42 (25.8)	6 (15.4)	3 (30.0)
Acneiform rash	41 (19.3)	38 (23.3)	3 (7.7)	0 (0.0)
Papulopustular rash	14 (6.6)	12 (7.4)	2 (5.1)	0 (0.0)
Erythema multiforme	7 (3.3)	7 (4.3)	0 (0.0)	0 (0.0)
Pruritus	16 (7.5)	12 (7.4)	3 (7.7)	1 (10.0)
Nail disorders				
Paronychia	16 (7.5)	12 (7.4)	4 (10.3)	0 (0.0)
Hair disorders				
Alopecia	1 (0.5)	1 (0.6)	0 (0.0)	0 (0.0)
Hypertrichosis	1 (0.5)	1 (0.6)	0 (0.0)	0 (0.0)
Mucositis	25 (11.8)	24 (14.7)	1 (2.6)	0 (0.0)
Gastrointestinal toxicities				
Diarrhea	43 (20.3)	34 (20.9)	8 (20.5)	1 (10)
Anorexia	10 (4.7)	9 (5.5)	1 (2.6)	0 (0.0)
Nausea	2 (0.9)	2 (1.2)	0 (0.0)	0 (0.0)
Vomiting	4 (1.9)	4 (2.5)	0 (0.0)	0 (0.0)
Others				
Liver injury	1 (0.5)	1 (0.6)	0 (0.0)	0 (0.0)
Dry eye	1 (0.5)	0 (0.0)	1 (2.6)	0 (0.0)
Dry lip	1 (0.5)	0 (0.0)	1 (2.6)	0 (0.0)
Neuropathy	1 (0.5)	1 (0.6)	0 (0.0)	0 (0.0)
Stomatitis	1 (0.5)	1 (0.6)	0 (0.0)	0 (0.0)
Duration (month)				
<1	98 (46.2)	84 (51.5)	11 (28.2)	3 (30.0)
1–3	60 (28.3)	47 (28.8)	12 (30.8)	1 (10.0)
>3–6	14 (6.6)	11 (6.7)	1 (2.6)	2 (20.0)
>6–12	5 (2.4)	3 (1.8)	2 (5.1)	0 (0.0)

**Table 3:** Grading severity of adverse events categorized by treatment episodes (*n* = 212).

Adverse Events	Incidence, *n* (%)		
	Overall	Grades 1–2	≥Grade 3
Cutaneous toxicities			
Rash	65 (30.7)	61 (93.8)	4 (6.2)
Xerosis	51 (24.1)	48 (94.1)	3 (5.9)
Acneiform rash	41 (19.3)	37 (90.2)	4 (9.8)
Papulopustular rash	14 (6.6)	14 (100.0)	0 (0.0)
Erythema multiforme	7 (3.3)	7 (100.0)	0 (0.0)
Pruritus	16 (7.5)	16 (100.0)	0 (0.0)
Nail disorders			
Paronychia	16 (7.5)	15 (93.8)	1 (6.3)
Hair disorders			
Alopecia	1 (0.5)	1 (100.0)	0 (0.0)
Hypertrichosis	1 (0.5)	1 (100.0)	0 (0.0)
Mucositis	25 (11.8)	23 (92.0)	2 (8.0)
Gastrointestinal toxicities			
Diarrhea	43 (20.3)	38 (88.4)	4 (9.3)
Nausea	2 (0.9)	2 (100.0)	0 (0.0)
Anorexia	10 (4.7)	10 (100.0)	0 (0.0)
Vomiting	4 (1.9)	4 (100.0)	0 (0.0)
Others			
Liver injury	1 (0.5)	1 (100.0)	0 (0.0)
Dry eye	1 (0.5)	1 (100.0)	0 (0.0)
Dry lip	1 (0.5)	1 (100.0)	0 (0.0)
Neuropathy	1 (0.5)	1 (100.0)	0 (0.0)
Stomatitis	1 (0.5)	1 (100.0)	0 (0.0)

**Table 4:** Adverse events and tumor response of EGFR-TKIs treatment episodes.

	Tumor Response, n (%)		*p*-Value
	Objective Response (*n* = 100)	Non-Response (*n* = 66)	
Cutaneous toxicities			
Rash	30 (30.0)	21 (31.8)	0.804
Xerosis	28 (28.0)	15 (22.7)	0.448
Acneiform rash	19 (19.0)	15 (22.7)	0.560
Papulopustular rash	9 (9.0)	4 (6.1)	0.490
Erythema multiforme	4 (4.0)	1 (1.5)	0.649 ^a^
Pruritus	12 (12.0)	1 (1.5)	0.014
Nail disorders			
Paronychia	8 (8.0)	4 (6.1)	0.765 ^a^
Hair disorders			
Alopecia	1 (1.0)	0 (0.0)	1.000 ^a^
Hypertrichosis	1 (1.0)	0 (0.0)	1.000 ^a^
Mucositis	9 (9.0)	10 (15.2)	0.223
Gastrointestinal toxicities			
Diarrhea	17 (17.0)	14 (21.2)	0.496
Nausea	0 (0.0)	2 (3.0)	0.157 ^a^
Anorexia	4 (4.0)	3 (4.5)	1.000 ^a^
Vomiting	2 (2.0)	2 (3.0)	0.650 ^a^
Others			
Liver injury	0 (0.0)	1 (1.5)	0.398 ^a^
Dry eye	0 (0.0)	1 (1.5)	0.398 ^a^
Dry lip	1 (1.0)	0 (0.0)	1.000 ^a^
Neuropathy	0 (0.0)	1 (1.5)	0.389 ^a^
Stomatitis	1 (1.0)	0 (0.0)	1.000 ^a^
Number of events			
0	17 (17.0)	11(16.7)	0.515
1	43 (43.0)	23 (34.8)	
≥2	40 (40.0)	32 (48.5)	

^a^ = Fisher’s exact test.

**Table 5:** Multivariable analysis of factors associated with objective response.

Variable	aOR (95% CI)	*p*-Value
Treatment line	0.27 (0.10–0.68)	0.006
Rash	0.89 (0.42–1.88)	0.753
Acneiform rash	0.79 (0.34–1.84)	0.580
Papulopustular rash	1.63 (0.45–5.94)	0.457
Pruritus	8.26 (1.00–67.75)	0.049
Paronychia	1.42 (0.38–5.38)	0.605
Diarrhea	0.81 (0.35–1.83)	0.607

## Data Availability

The data that support the findings of this study are available on request from the corresponding author.

## Abbreviations

The following abbreviations are used in this manuscript:

EGFR-TKIs	epidermal growth factor receptor tyrosine kinase inhibitors
NSCLC	non-small cell lung cancer
AEs	adverse events
ASCO	American Society of Clinical Oncology
ESMO	European Society for Medical Oncology
NCCN	National Comprehensive Cancer Network
